# Presence of Meaning Mediates the Relationship Between Meaning Search and Outcome: A Cross-Cultural Study

**DOI:** 10.1093/geroni/igaa057.986

**Published:** 2020-12-16

**Authors:** Nicole Long Ki Fung, Helene Fung

**Affiliations:** The Chinese University of Hong Kong, Hong Kong, China

## Abstract

Search for meaning (SFM) is associated with many well-being measures. The mechanism behind remains unclear. This study explores presence of meaning (POM) as a mediator to explain the association. While dialectical thinking in Eastern cultures values both process and outcome, oppositional thinking in Western cultures makes the two opposing. Since dialectical thinking increases with age, we hypothesize that with increased age, SFM is associated with POM more positively (less negatively). This heightened POM results in better well-being. We surveyed 2014 participants (aged 18-96, Mage= 55.6) in Eastern cultures: Hong Kong and Taiwan; Western cultures: Germany, United States and the Czech Republic. In Eastern cultures, SFM was positively associated with POM and life satisfaction. POM partially mediated the relationship between SFM and life satisfaction (b=0.328, p<.001). With age, SFM was associated more positively with POM and life satisfaction (b=0.009, p<.001). While POM partially mediated the relationship in younger adults (b=0.162, p<.001), full mediation was found in older adults (b=0.451, p<.001). In Western cultures, SFM was negatively associated with POM and life satisfaction. POM partially mediated the relationship between SFM and life satisfaction (b=-0.120, p<.001). With age, the negative association of SFM with POM and life satisfaction was attenuated (b=0.002, p<.001). These finding suggested that SFM becomes more beneficial to older adults across culture via establishing POM. Identifying factors that facilitate the process of achieving meaning through searching is therefore important.

